# Mismatch Negativity (MMN) in Freely-Moving Rats with Several Experimental Controls

**DOI:** 10.1371/journal.pone.0110892

**Published:** 2014-10-21

**Authors:** Lauren Harms, W. Ross Fulham, Juanita Todd, Timothy W. Budd, Michael Hunter, Crystal Meehan, Markku Penttonen, Ulrich Schall, Katerina Zavitsanou, Deborah M. Hodgson, Patricia T. Michie

**Affiliations:** 1 School of Psychology, University of Newcastle, Callaghan, NSW, Australia; 2 Priority Centre for Translational Neuroscience and Mental Health Research, University of Newcastle, Newcastle, NSW, Australia; 3 Schizophrenia Research Institute, Darlinghurst, NSW, Australia; 4 Hunter Medical Research Institute, Newcastle, NSW, Australia; 5 School of Medicine and Public Health, University of Newcastle, Callaghan, NSW, Australia; 6 Department of Psychology, University of Jyvaskyla, Jyvaskyla, Finland; 7 School of Psychiatry, Faculty of Medicine, University of New South Wales, Sydney, NSW, Australia; 8 Neuroscience Research Australia, Randwick, NSW, Australia; University of Salamanca- Institute for Neuroscience of Castille and Leon and Medical School, Spain

## Abstract

Mismatch negativity (MMN) is a scalp-recorded electrical potential that occurs in humans in response to an auditory stimulus that defies previously established patterns of regularity. MMN amplitude is reduced in people with schizophrenia. In this study, we aimed to develop a robust and replicable rat model of MMN, as a platform for a more thorough understanding of the neurobiology underlying MMN. One of the major concerns for animal models of MMN is whether the rodent brain is capable of producing a human-like MMN, which is not a consequence of neural adaptation to repetitive stimuli. We therefore tested several methods that have been used to control for adaptation and differential exogenous responses to stimuli within the oddball paradigm. Epidural electroencephalographic electrodes were surgically implanted over different cortical locations in adult rats. Encephalographic data were recorded using wireless telemetry while the freely-moving rats were presented with auditory oddball stimuli to assess mismatch responses. Three control sequences were utilized: the *flip-flop* control was used to control for differential responses to the physical characteristics of standards and deviants; the *many standards* control was used to control for differential adaptation, as was the *cascade* control. Both adaptation and adaptation-independent deviance detection were observed for high frequency (pitch), but not low frequency deviants. In addition, the *many standards* control method was found to be the optimal method for observing both adaptation effects and adaptation-independent mismatch responses in rats. Inconclusive results arose from the *cascade* control design as it is not yet clear whether rats can encode the complex pattern present in the control sequence. These data contribute to a growing body of evidence supporting the hypothesis that rat brain is indeed capable of exhibiting human-like MMN, and that the rat model is a viable platform for the further investigation of the MMN and its associated neurobiology.

## Introduction

One of the most commonly reported and replicable electrophysiological abnormalities observed in people with schizophrenia is the reduction in the amplitude of the mismatch negativity (MMN) in response to deviations in the acoustic environment [1]–[3]. In adult humans, MMN is evident as a negative shift in the auditory event-related potential (ERP) elicited by a rare, unexpected stimulus (the *deviant*) when it interrupts a train of common, expected stimuli (the *standards*), and typically occurs 100–200 ms after stimulus onset [4], [5]. A meta-analysis reported that persons with schizophrenia exhibit reductions in the size of the MMN with an overall effect size of 0.99 [3]. MMN responses can be observed in different states of consciousness and in the absence of attention to the stimuli, leading to its characterisation as an automatic, pre-attentive process [4]. MMN is primarily generated in the auditory cortex, with some contribution from the frontal cortex and other areas [6], [7]. It has been observed in neural activity measured using electroencephalography (EEG) [8], magnetoencephalography [9] and optical imaging [10]. MMN is typically measured using *oddball* sequences of auditory stimuli, in which a repeated train of standards is unexpectedly interrupted by a low-probability deviant. MMN is commonly elicited by presenting deviants that differ from the standards in some simple characteristic feature, such as frequency or duration [8].

**Figure 1:**
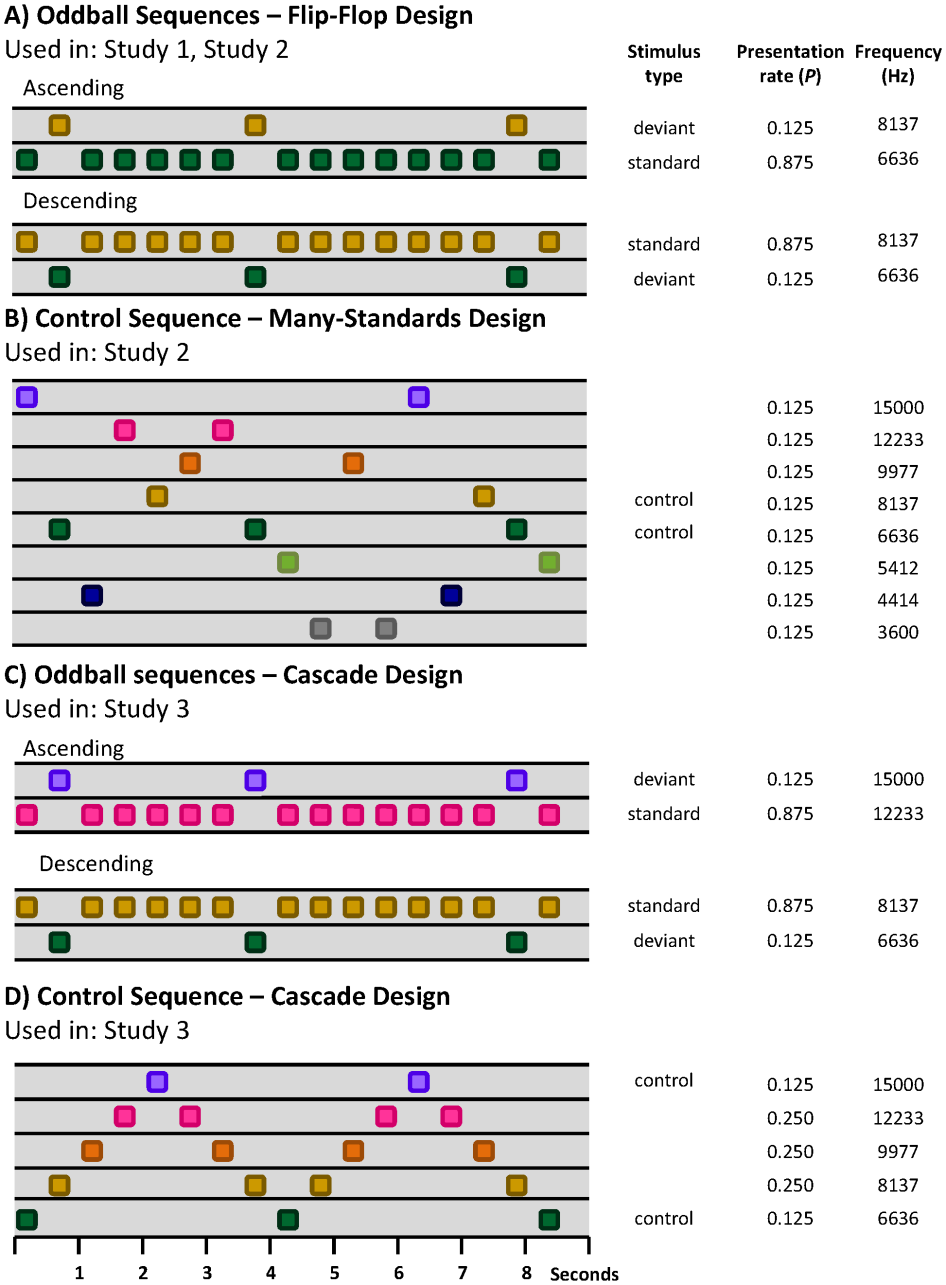
Control sequence designs used in the current investigation. (**A**) Both Study 1 and Study 2 used a flip-flop design for oddball sequences. This design allows for the comparison of the response to a stimulus when it is a rare, unexpected deviant to the same tone when it is a common, expected standard, controlling for differences in responses to the physical characteristics of the stimuli, but not for differential adaptation. (**B**) The many-standards control sequence was used in Study 2 to test responses to stimuli that would have prompted the same level of adaptation as the deviant stimulus. Comparing the responses to the deviant used in the oddball sequence, where it defies established stimulus regularity predictions, to the control in the many-standards sequence, which does not defy regularity predictions (there is no established regularity) yields a measure of adaptation-independent deviance detection. Comparing the responses to the control (presented rarely) in the many-standards sequence to the standard (presented often) in the oddball sequence yields a measure of adaptation to a rare stimulus vs. a common stimulus. (**C**) The cascade sequence designs were used for Study 3. The oddball sequences are similar to those used in Study 1 and Study 2, except that a flip-flop design was not used, and the stimuli presented in the ascending and descending sequences are respectively on the upper and lower end of the range of sequences used in the control sequence. (**D**) The control sequence (like the many-standards sequence) presents the stimuli used as deviants in the oddball sequences at the same probability as they are when deviants in a context where they do not defy established patterns in regularity, thus enabling the control of differential adaptation.

In recent years, the MMN research community has begun to focus on developing animal models of MMN, in order to investigate the neurobiological mechanisms underlying the MMN, such as the role of specific neurotransmitter systems, contributions from different cortical layers or brain regions to the surface potential, and relationship to upstream effects that appear to be related to MMN such as stimulus specific adaptation (SSA) [11], [12]. Several models in rats, mice and non-human primates have been studied with varied results (for detailed review, [2]). There are two important factors that need to be examined and controlled when identifying an animal homologue of the human MMN: first is the possibility of differential responses to the physical characteristics of the stimuli used as the standards and deviants. This is addressed in *flip-flop* sequences, where two oddball sequences are presented with the identity of the standard and deviant reversed (e.g. a particular tone is the deviant in one sequence and the standard in the other). This permits the response to a stimulus when it is a deviant to be compared to the same stimulus when it is a standard (Figure 1A). The second factor is the role of *adaptation* versus *‘true’ deviance detection*, which, within some theoretical frameworks, is considered to be a memory-based or a predictive coding error signal [13]–[15]. Several studies in both humans and animals have shown that with repeated exposure to a stimulus, the neural populations responding to that stimulus undergo *adaptation*, in which their responses are dampened with higher probabilities of stimulation [12], [16]–[23]. This means that a larger response to a deviant stimulus may simply be due to lower levels of adaptation of neural populations responding to a rare stimulus (the deviant) compared to a frequent stimulus (the standard). Note that while the term *adaptation* is used here, other terms are often used to describe similar, but not exactly synonymous phenomena (the reduction of a response to a stimulus with repeated exposure), such as habituation, refractoriness, and stimulus-specific adaptation. This is addressed in several studies by using a *many-standards control* sequence (Figure 1B). In this sequence, the deviant tone from the oddball sequence is presented with the same probability as it is presented within the oddball sequence, but it is nested within many other equally-probable tones. The tones are presented pseudo-randomly (without repetition) so that no pattern of regularity is established. This lack of regularity ensures that no specific ‘prediction’ is set that can be violated. Comparing the response to the same physical stimulus when it is a deviant within the oddball sequence, to when it is the control stimulus within the many standards sequence, provides a measure of the adaptation-independent comparison process contribution that is thought to underlie MMN.

Using these approaches, MMN in humans has been found to comprise two ‘elements’ after controlling for the physical characteristics of the stimulus using the flip-flop control method: an adaptation element (‘sensorial’ element, represented by the difference between the rare control stimulus and the common standard stimulus), and a prediction error-like element (‘cognitive’ element, represented by the difference between the rare control stimulus and the rare deviant stimulus) [19], [20], [24]–[26]. While this feature of MMN has several designations: memory-based MMN [27], [28], prediction error [29], cognitive (versus sensory) MMN [19], [20], and deviance detection [21], [30]–[32] to name a few, for the purposes of clarity in the remainder of the paper, *adaptation-independence* and/or *deviance detection* will be used to describe the element of MMN that remains when adaptation is controlled for.

While the two elements comprising MMN (adaptation and adaptation-independent deviance detection) are not commonly disentangled in human studies (for exceptions, [19], [20]), it is important for animal models to test for both. This is because a) it is unknown whether a given species is capable of generating an adaptation-independent response as humans do, and b) these two elements likely have diverging neural mechanisms and signatures. If the aim of establishing an animal model of MMN is to investigate the underlying neural mechanisms of MMN, then the MMN elements being investigated should be identified.

The many standards control sequence, as mentioned, controls for the effects of stimulus presentation probability so that differential adaptation can be observed in the absence of the established models of regularities. However, this control has been subject to two criticisms [33]. First, it may be overly conservative, because the variety (usually frequency range) of stimuli presented in the many standards sequence is substantially larger than that in the oddball paradigm. It has been demonstrated using local field potential and multiunit activity recordings that many standards sequences using a broad range of frequencies produce larger responses than those from narrow sequences, regardless of presentation rate [21]. This would indicate that potentially, the control response in the many standards sequence is not affected as much by adaptation and therefore is increased in amplitude. However, the deviant response from the oddball sequence (containing a narrow range of stimuli) would presumably undergo *more* adaptation than the control response, and consequently be reduced in size. This possible imbalance in the amount of adaption undergone by the control and deviant responses could result in an underestimation of the difference between the control and deviant responses (deviance detection). Second, in the oddball paradigm, deviants are presented within a repetitive, predictable sequence, but no such repetition is established within the many standards control sequence. To avoid these issues, a cascade control sequence has been proposed [33]. In this method, firstly, there are a small number, nominally five, stimuli that vary from low to high frequency, with the highest frequency stimulus corresponding to the deviant, and the second highest frequency stimulus corresponding to the standard within an ascending oddball sequence (Figure 1C,D). Secondly, the stimuli are presented in a regular pattern from low to high frequency, then back down to low frequency, repetitively (Figure 1D). The high-frequency stimulus at the upper extreme of the stimulus range is used as a control for high-frequency deviants in the ascending oddball task. An equivalent sequence can be adapted for low-frequency deviants in a descending oddball sequence. Within the cascade sequence, the variety of stimuli presented is more comparable to that in the oddball task, and the control stimulus is always preceded by a stimulus that is physically identical to the standard within the oddball task. This can improve the estimation of adaptation effects. In addition, the cascade control incorporates a background regularity, albeit a more complex one than oddball sequences, but where the occurrence of the high (and low) frequency tones at the extremes of the cascade sequence are predictable, in contrast to the equivalent high (and low) frequencies of oddball sequences. Therefore, the cascade control provides the opportunity to observe adaptation-independent deviance detection in the context of unpredictable deviants vs. predictable deviants, assuming of course that the rat brain is able to model the regularity of the cascade sequence.

In a recent review of animal models of MMN [2], several important trends were identified. First, mismatch responses (MMR) in animals typically occur earlier than in humans, likely due to the smaller brain size. Second, the difference between the deviant and the standard can be either negative or positive in polarity (positive shifts are far more common in recordings in anaesthetised animals, particularly when anaesthetised with urethane). Finally, deviance detection that is independent of the effects of differential adaptation is rarely observable in small-field recordings (e.g. local field potentials, multiunit activity) from primary auditory cortex [17], [18], [21], but are more often observed with epidural electrodes [28], [29], [34]–[38]. This is in agreement with emerging studies suggesting that non-primary areas of the auditory cortex are involved in adaptation-independent MMN [20]. Indeed a recent study has demonstrated that while non-MMN components of the auditory ERP are localised to primary (core) auditory cortical areas and can be tonotopically mapped, adaptation-independent MMN is highly distributed over the auditory cortex, including secondary auditory ‘belt’ regions and are not tonotopically localised [32]. All of the studies that instituted a control method for adaptation effects used either a deviant-alone control (another method for controlling for adaptation) or the many standards control sequence. To our knowledge, the cascade control method has not been tested in an animal model thus far.

Our laboratory has previously published one of the aforementioned animal model studies [35], in which adaptation-independent deviance detection was observed in awake rats to deviants in stimulus frequency. These were only observed for high-frequency deviants (3600 Hz), not low-frequency deviants (2500 Hz), in agreement with similar findings in anaesthetised rats [28], indicating a possible enhanced salience for increments in frequency, compared to decrements. Another possible reason for why rats in our previous study exhibited deviance detection to high but not low frequency stimuli could be the frequency of the tones that were tested (2500 and 3600 Hz), which were at the lower end of the rats' frequency sensitivity [39], [40]. Examining whether deviance detection can be elicited to higher-frequency tones that are closer to the peak of rats' frequency sensitivity (e.g. ∼16000 Hz) will determine if the preference for high-frequency deviants was an artefact resulting from the use of low frequency sounds.

Many previous studies in animal models have only investigated MMRs in single locations, typically over auditory cortex. However, it is possible that MMRs in rats are more readily observed at other locations depending upon the orientation and location of generators. In humans for example, the major generators of MMN are located in auditory cortex, yet the largest response is seen over frontal areas even though there may be only a small contribution from frontal generators [7], [41]. Therefore, an examination of the effect of recording location on the amplitude of MMRs is warranted.

In the current investigation, we aimed to replicate our laboratory's previous evidence of adaptation-independent deviance detection in the rat, with the overall aim of determining which conditions produce the most robust adaptation responses and adaptation-independent deviance detection responses. In Study 1, we used the same recording system previously used in our laboratory [35], to determine if MMRs are produced to tones of higher frequencies (closer to the rats' peak frequency sensitivity). In Study 2, we used a new system allowing multichannel recordings, to characterize the morphology at different locations of adaptation and adaptation-independent deviance detection using the many standards control. Finally, in Study 3, we investigated the utility of the cascade control method for recording MMRs in rats.

## Methods

### 2.1 Ethics Statement

All experiments were performed under strict adherence to the National Health and Medical Research Council's Australian code of practice for the care and use of animals for scientific purposes and were approved by the University of Newcastle's Animal Care and Ethics Committee (Approval number A-2009-108). Surgical procedures were performed under well-maintained anaesthesia and all efforts were made to reduce the number of animals used and alleviate pain and discomfort following surgery through use of analgesics.

### 2.2 Animals and Surgery

#### 2.2.1 Study 1

Nine male Wistar rats (sourced from the University of Newcastle's Central Animal House) were used for Study 1. All rats were on a 12 h light/dark cycle with lights on at 06:30 h. The surgery was performed when the animals were on average 96 days old (89–111 days old). The average weight of the animals was 456.9 g (381–513 g) on the day of surgery. Animals were anaesthetised with fentanyl (300 µg/kg i.p.) and medetomidine (300 µg/kg i.p.), and/or isoflurane and the rat was fixed onto a stereotaxic frame (Stoelting, IL, USA) and placed on a heating pad during surgery. A battery operated biotelemetric radiotransmitter (model # TA11CA-F40, Data Sciences International, St. Paul, MN, USA) was implanted in the peritoneal cavity. Insulated biopotential leads from the transmitter were passed subcutaneously to the base of the skull. The skin over the skull was exposed and 2 small burr holes were drilled in the skull, one hole for the active electrode over the right auditory cortex (4.5 mm posterior to Bregma and 3.5 mm lateral to the midline) and the other for the reference electrode in the left cerebellum (2 mm posterior to the lambda and 2 mm lateral to the midline). These locations are based on previous research demonstrating MMN-like epidural responses in the rat [37]. The leads were fixed with dental acrylic. Carprofen (5 mg/kg s.c.) and buprenorphine (0.05 mg/kg s.c.) were administered pre-operatively as analgesics. The animals were allowed to recover for at least 6 days after surgery before the first ERP recordings.

Testing occurred within an experimental chamber covered with grounded copper mesh acting as a Faraday cage. The rat was placed in a partition (internal dimensions: length 23.5 cm, width 12.0 cm, height 24.0 cm) within the experimental chamber. EEGs were recorded using custom acquisition software written in LabVIEW (version 8.2.1). Two channels of data were continuously digitised (1000 Hz): a single EEG channel, and an analogue trigger pulse generated by the PC sound card in parallel with the auditory stimulus. Stimulus event codes were logged with the EEG data. The bandwidth of the data acquisition system was 0.2–150 Hz and the input voltage range was ±10 mV.

#### 2.2.2 Studies 2 and 3

Eighteen male Wistar rats were used for Study 2, 15 of which were also used for Study 3. These rats were on a 12 h light/dark cycle with lights on at 00:00 h (midnight) and were used as controls for another study investigating the role of developmental exposure to immune activation on electrophysiological measures. Seven female Wistar rats (sourced from the University of Newcastle's Central Animal House) were time-mated with three male Wistar breeders. The day of positive sperm detection was designated as gestational day (GD) 0. Four pregnant females were injected with saline on GD10 and three were injected with saline on GD19, resulting in nine male offspring exposed to prenatal saline injection at GD10 and nine at GD19, all of which were used for Study 2. For Study 3, only eight rats exposed to GD10 injection and seven exposed at GD19 were used. Pregnant females were anesthetised with isoflurane, and given an intravenous administration (via the lateral tail vein) of 0.1 M phosphate buffered saline (at 1 mL/kg body weight).

The surgery to implant electrodes was performed on the male offspring of these pregnant animals when they were, on average, 108 days old (76–137 days) and weighed on average 481.76 g (370–593 g). Rats were anaesthetised with isoflurane, placed on a heating pad, and fixed to a stereotaxic frame (Stoelting, IL, USA). The dorsal surface of the skull from +4.00 mm to −12.00 mm relative to Bregma and 4–5 mm lateral from the midline was exposed and the periosteum was removed. A custom-made electrode connector was implanted onto the rat's skull. The connector consisted of a 10-pin male-female socket (BD075-10-A-1-L-D from Global Connector Technology, Lawrence, MA, USA), with the pins soldered to magnet wire (8057 from Belden, St Louis, MO, USA) and embedded in epoxy resin (RS 1991402, RS Components, Sydney, Australia). Seven wires from the connector were soldered to stainless steel screws (B002SG89S4, Amazon Supply, USA). Seven 0.9 mm burr holes were made into the skull of the rat, penetrating all the way through the skull, but not through the dura. The screw electrodes were implanted into these holes until they were fixed in place. Five screws were used as recording electrodes and were placed above the left and right auditory cortices (LAC and RAC, 5.00 mm posterior to Bregma and 4.00 mm lateral to the midline), the left and right frontal cortices (LFC and RFC, 2.00 mm anterior to Bregma and 2.00 mm lateral to the midline), and a location to the left of the midline (LML, 3.50 mm posterior to Bregma and 1.00 mm left of the midline). The ground screw was placed over the right posterior cortex (2.0 0 mm anterior to Lambda and 2.50 mm right of the midline), and the reference screw over the cerebellum (1.00 mm posterior to Lambda and 1.00 mm to the right of the midline). The wire connecting the screw electrodes to the connector was wound around their respective screws and the wires, screws and socket were fixed to the animal's head using dental cement (Dentsply, Mount Waverly, VIC, Australia). Carprofen (5 mg/kg s.c.) and buprenorphine (0.05 mg/kg s.c.) were administered pre-operatively as analgesics. The animals were allowed to recover for at least 4 days after surgery before the first ERP recordings.

Immediately prior to testing, a wireless telemetric 8-channel headstage from Multi Channel Systems (Reutlingen, Germany) was connected to a battery using reusable adhesive, and then attached to the electrode connector previously implanted on the rat's head. Testing occurred within an expanded PVC sound-attenuating chamber (ENV-018V, Med Associates, St. Albans, VT USA) with the interior covered with sound-absorbing foam. The awake rat was placed in a 32 cm diameter plastic bucket, containing pressed-paper bedding, where it was free to roam. EEG data were recorded using Multi Channel Systems MCRack software. Each channel of EEG data was digitised at 200 0 Hz (high pass filter 0.1 Hz; low pass filter 5000 Hz; voltage range ±12.4 mV). Event code markers and a trigger pulse generated by the sound card in parallel with the auditory stimuli were recorded as digital signals at the same sampling rate.

### 2.3 Sound Generation

#### 2.3.1 Study 1

Auditory stimuli were generated with a custom program written in Presentation (version 14.1, Neurobehavioral Systems, Inc.), amplified and delivered through a speaker (50 Hz–19000 Hz frequency response) mounted at an approximate height of 1 m above the floor of the experimental chamber. Sound intensity was calibrated with a sound meter (Brüel & Kjær Model 2260) using a linear weighting to an average of 78 dB_L_ SPL across locations within the chamber for the sounds in the 6636 and 8137 Hz range used in this study.

#### 2.3.2 Studies 2 and 3

Auditory stimuli were generated with a custom program written in Presentation (version 14.1, Neurobehavioral Systems, Inc.), amplified and delivered through a speaker (1 kHz–30 kHz frequency response) mounted at an approximate height of 50 cm above the floor of the experimental chamber. Sound intensity was calibrated with a sound meter (Brüel & Kjær Model 2260) using a linear weighting to an average of 70 dB_L_ SPL across locations within the chamber for the sounds in the 3600 and 15000 Hz range used in these studies.

### 2.4 Experiment Design and Stimuli

#### 2.4.1 Study 1 – Flip-flop Control

Rats were tested for one half-hour session each day, for three days. The awake rat was placed in the experimental chamber for 15 min before each session to acclimatise. Each session consisted of an ascending and a descending oddball sequence separated by a 3 min break. The order of the two sequences alternated for each rat across test sessions, and was balanced across rats.

Two sequences were presented in Study 1. These were oddball sequences where the roles of the deviant and standard were reversed (flip-flop condition) resulting in either an ascending deviant sequence (low frequency standard and high frequency deviant) or a descending deviant sequence (high frequency standard and low frequency deviant) (Figure 1A). In the ascending and descending oddball sequences, 87.5% of the tones were standards and 12.5% deviants. Previous findings have demonstrated that certain components of the deviant response were sensitive to the recent stimulus history of standards, in that it increased in amplitude as the number of preceding standard increased from 1 to 5 or more [35]. In order to maximise MMRs, the oddball sequences of Studies 1–3 reported here were designed to have at least 3 standards prior to each deviant. For all sequences, tones had a 10 ms rise and fall time and a stimulus onset asynchrony (SOA) of 500 ms. Two tones of 100 ms duration were used: a low frequency tone of 6636 Hz and a high frequency tone of 8137 Hz, equivalent to a 0.29 octave difference or normalised frequency difference (or Δf) of *(f_2_−f_1_)/(f_2_×f_1_)^1/2^* = 0.20 where *f_1_* = 6636 Hz and *f_2_* = 8137 Hz [12]. Each of the sequences consisted of 1600 tones and ran for 13.33 min.

#### 2.4.2 Study 2 – Many-Standards Control

Rats were tested for MMRs on one 62 min session a day for three days, and were exposed to three different testing orders for each of the three days. The rat was placed in the experimental chamber with bedding for 5 min before each session to acclimatise. The rat did not have access to food or water during the session but was free to explore the testing chamber during the recordings. On each of the three testing days, the rat was also tested in separate sessions on two other auditory paradigms that are not reported here.

Each session in Study 2 consisted of four types of sequences each presented twice, resulting in eight sequences per session. Two of the four types of sequence were the ascending and descending oddball sequences described for Study 1 (Figure 1A). The other sequences were many-standards control sequences in which tones equivalent to the deviants in the ascending and descending oddball sequences were presented at the same probability as in the oddball sequences (12.5%) but randomly interspersed with six other tones (also presented at 12.5%), ensuring that a pattern of regularity in the auditory stimuli was not established [11], [24] (Figure 1B).

The two many-standards control sequences were subtly different in order to accommodate the pseudo-random stimulus orders within the ascending and descending oddball sequences. For all sequences, tones had a 10 ms rise and fall time, a duration of 100 ms and a SOA of 500 ms. Eight frequencies (each of 100 ms duration) differing on a logarithmic scale were presented: 3600 Hz, 4414 Hz, 5412 Hz, 6636 Hz (equivalent to oddball low frequency deviant), 8137 Hz (equivalent to high frequency oddball deviant), 9977 Hz, 12233 Hz and 15000 Hz. In the first of the control sequences, the 8137 Hz stimulus was presented in exactly the same temporal location (relative to the beginning of the sequence) as in the ascending oddball deviant sequence. In the second of the control sequences, the 6636 Hz stimulus was presented in the same temporal location as in the descending oddball deviant sequence, but neither of the sequences controlled for the tone preceding the deviant. The remaining tones were presented in pseudorandom order except that no tone was ever repeated. In order to avoid the possibility of an MMN being elicited by tones at the extremes of a range for either the frequency control conditions, known as the extreme substandard effect [24], [26], [42], the standard and deviant used in the ascending and descending sequences were the fourth and the fifth highest frequencies (Figure 1B).

Within each session, sequences were presented in one of four orders, and repeated in that same order. Blocks of sequences began with one oddball sequence, followed by the two control sequences (with two order combinations), and ending with the other oddball sequence. Within a block, sequences were separated by 1 min silent breaks and a 3 min silent break separated the two blocks. Each sequence contained 800 stimuli and ran for 6.67 minutes, and each session ran for 62 min.

#### 2.4.3 Study 3 – Cascade control

The same animals used for Study 2 were also used in Study 3. Rats were tested for one recording session using the cascade control sequences. The rat was placed in the experimental chamber for 5 min before the commencement of the session and was free to explore during recordings. Three types of sequence were presented, and similar to Study 2, each sequence consisted of 800 tones, each played with 100 ms duration, 10 ms rise and fall time and SOA of 500 ms. Similar to Studies 1 and 2, Sequences 1 and 2 were ascending and descending oddball sequences (Figure 1C). The ascending sequence consisted of a low-frequency standard (12233 Hz, 87.5%) and a high frequency deviant (15000 Hz, 12.5%), and the descending sequence consisted of a high-frequency standard (8137 Hz, 87.5%) and a low-frequency deviant (6636 Hz, 12.5%). Sequence 3 was a cascade control sequence (Figure 1D). Five tones were presented in this sequence: 6636 Hz, 8137 Hz, 9977 Hz, 12233 Hz and 15000 Hz, played in order from lowest frequency to highest frequency, back to lowest frequency in a ‘cascading’ order, similar to Ruhnau et al. [33]. In this sequence, the two tones used as deviants in ascending and descending oddball sequences are presented with the same probability as in the oddball sequences (12.5%), whereas the other tones are presented at a probability of 25%. Within a session, sequences were presented in one of two orders: either 1) Ascending, Control, Descending; or 2) Descending, Control, Ascending. Each of these blocks was presented twice within a session with a 3 min silent break between the two blocks and 1 min silent breaks between each sequence within a block. The total session time was approximately 49 min.

### 2.5 Data Extraction

Data processing was performed off-line with EEGDisplay 6.3.12 [43]. Intervals of gross artefacts in the continuous EEG record were excluded using an automated algorithm that rejected signals exceeding ±1400 µV. Epochs were extracted from the continuous EEG consisting of a 100 ms pre-stimulus baseline and a 400 ms post-stimulus interval. The first 25 tones at the start of each tone sequence were excluded from analysis to allow for transitory effects associated with switching between different types of sequences or the beginning of the session. Within oddball sequences, the first standard following each deviant was excluded from the analysis to allow for recovery of a stable response to standards. Following these pre-processing steps, epochs were averaged off-line for each animal and session separately and ERPs extracted for each of the stimulus types, including the responses to deviants and standards, as well as their respective controls for each of the studies. ERPs were baseline corrected over a 50 ms pre-stimulus interval for Study 1 and a 100 ms pre-stimulus interval for Studies 2 and 3.

The ERPs recorded in these studies exhibited distinct components over the first 200 ms, although the amplitudes of these components differed according to the type and frequency of the stimulus. For Study 1, they were characterised by a negative component peaking at approximately 22 ms (denoted as N22), followed by a positive peak at 37 ms (P37) and a second broad negative component with a peak latency of approximately 60–100 ms (N80). For Studies 2 and 3 (using different EEG recording and acoustic delivery), the components were identified to occur slightly earlier with an additional early positive component being identified, and a clear separation of the broad late negative shift into two distinct peaks. The ERP was characterised by an initial positive peak at 13 ms (P13), a negative peak at 18 ms (N18), followed by a positive peak at 30 ms (P30) and a broad negative component with two discernible peaks from approximately 45–65 ms (N55) and 65–105 ms (N85).

**Figure 2:**
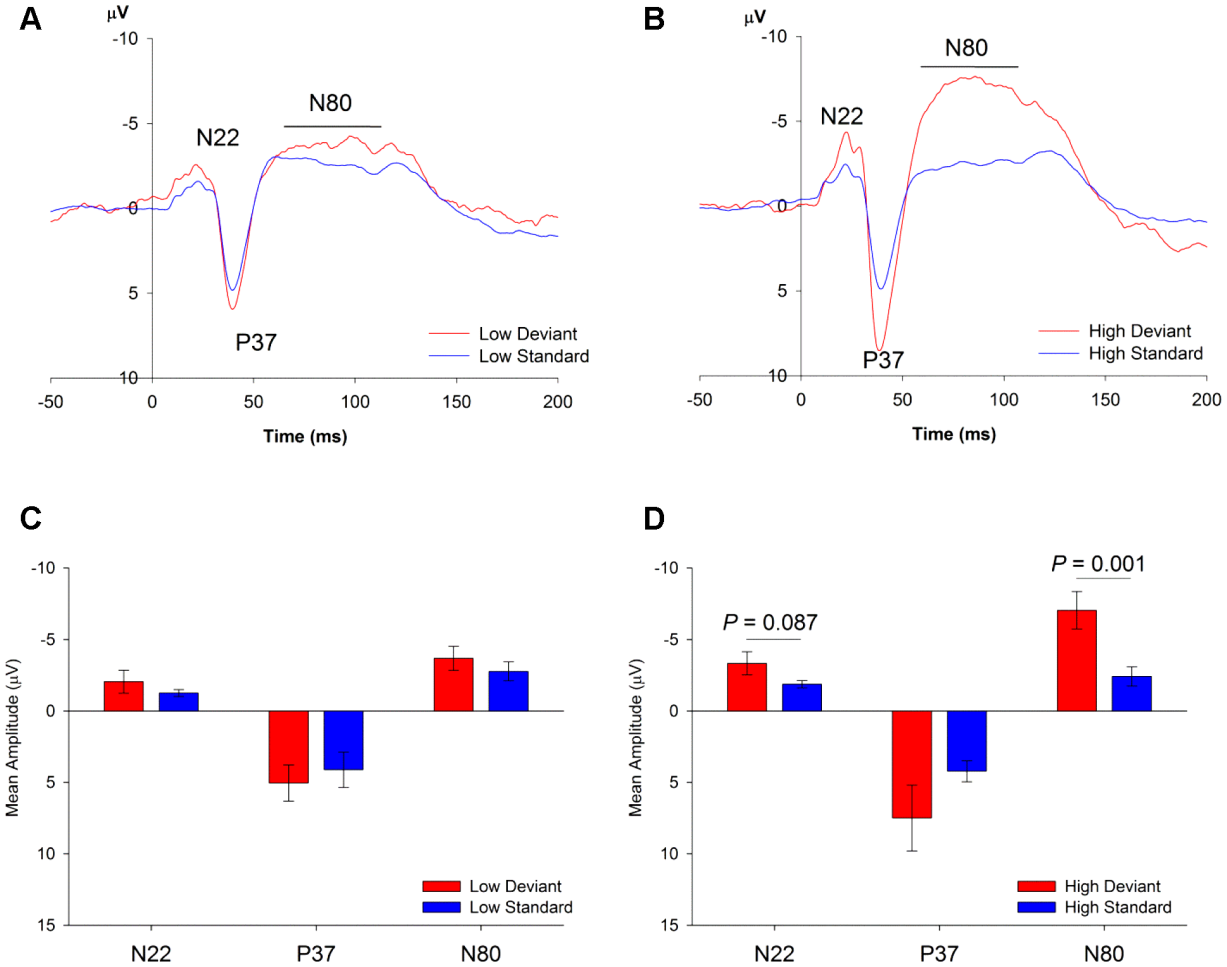
Rat ERPs in Study 1. (**A,B**) ERPs to the oddball deviant (red) and the standard (blue) for the low (**A**) and high (**B**) frequency stimuli. All stimuli show a similar pattern with the same components (N22, P37, N80): responses to deviants are larger in amplitude in comparison to standards. (**C,D**) Mean amplitudes (± standard error, SE) of N22, P37 and N80 generated by oddball deviants (red) and standards (blue), showing that responses to the deviant compared to the standard were larger for the N80 component (*P* = 0.001), and the N22 (although not significantly, *P* = 0.087).

For Study 1, three mean amplitude measures were extracted over latency windows corresponding to the ERP peaks: a 15 ms window from 15–30 ms for N22, an 8 ms window from 35–43 ms for P37 and a 40 ms window from 60–100 ms for N80. A wide window was used to assess N22 because there were relatively large individual differences in the latency of the peak, which was far more variable across animals than P37 (Figure 2). For Studies 2 and 3, five mean amplitude measures were extracted over the following latency windows: a 4 ms window from 11–15 ms (P13), a 7 ms window from 15–22 ms for N18, a 21 ms window from 22–43 ms for P30, a 23 ms window from 43.5–65.5 ms for N55 and a 40 ms window from 65.5–105.5 ms for N85.

### 2.6 Statistical Analysis

All analyses were performed controlling for stimulus identity. That is, only responses to stimuli of the same frequency were compared. For example, although the 8 kHz deviant in Study 2 was presented in an ascending oddball sequence with a 6 kHz standard, all analyses performed on the 8 kHz deviant involved comparisons with the 8 kHz standard (used in the descending oddball sequence) and the 8 kHz control (used in the many-standards control sequence). Therefore, for this study, when referring to a Deviant vs. Standard comparison, we do not refer to the Deviant and Standard tones within an individual sequence, rather we refer to the deviant tone of a certain frequency and its respective flip-flop controlled standard tone of the same frequency.

Mean amplitudes of the ERP components were analysed using Analysis of Variance (ANOVA) with one or more repeated measures factors depending upon the study. Within-subjects factors were *Stimulus Type* (Study 1: Deviant and Standard, Study 2: Deviant, Control and Standard, Study 3: Deviant and Control), Stimulus Frequency (Studies 1 and 2: 6636 Hz, 8137 Hz; Study 3: 6636 and 15000 Hz) and electrode location (in the case of Studies 2 and 3, left and right auditory cortices, LAC and RAC; left and right frontal cortices, LFC and RFC; and left of the midline, LML). Each ERP component was analysed separately. Gestational age of maternal treatment (for Studies 2 and 3 only) with saline was also used as a between-subjects factor to ensure that the different gestational day of treatment did not impact findings in this group of animals. In instances where sphericity was violated, Huynh-Feldt adjusted degrees of freedom were used to determine significance levels.

Given the large number of regions and components analysed in Study 2 and 3, significance levels for the first-pass, omnibus ANOVA were set at P<0.01, to reduce the likelihood of Type 1 errors. Once an effect was identified in this first ANOVA, follow-up ANOVAs and *post-hoc* comparisons used a significance level of P<0.05. *Post hoc* pairwise comparisons were made using Bonferroni correction and *P* values will be expressed as the Bonferroni-corrected value, *P_b_*. These pairwise comparisons were used to determine whether *oddball effects*, *deviance detection* or *adaptation* were present to a statistically significant degree. *Oddball effects* occur when the amplitude of the response to the deviant is significantly larger than that to the standard stimulus (i.e. more positive for positive components and more negative for negative components) and these were assessed in Studies 1 and 2 (designs in which deviants and standards of the same frequency were presented). This measure of MMN, while controlling for different stimulus frequencies, does not comprise a control for different levels of adaptation to the standard and deviant stimuli. Therefore, two other comparisons were made. In Studies 2 and 3, the amplitude response to the deviant was compared to the control to determine the level of *deviance detection*, and in Study 2, the amplitude response to the control was compared to the standard to determine the level of *adaptation*. The magnitude of these oddball, adaptation and deviance detection effects were expressed as effect sizes measured by Cohen's *d*.

#### 2.6.1 Incomplete data

In Studies 2 and 3, some animals did not have a complete dataset. This was caused by poor-quality, noisy EEG traces from particular electrodes that caused certain regions to be removed from analysis.

For Study 2, only two rats had incomplete data (both were missing data for three regions). These rats were removed from the analysis, resulting in a final sample size of 16 for Study 2.

For Study 3, on the other hand, there were a large number of animals with incomplete data: no regions produced usable data in all animals. A total of seven rats had incomplete data, but none were missing data for more than three regions. The data for Study 3 were initially analysed without the seven animals with incomplete data. However, removing so many from the analysis could result in a dramatic loss of useful information and reduced power. For instance, only one animal had missing data from each of the frontal cortex sites. Therefore, in order to still utilize the full dataset from Study 3, automatic imputation was used to impute the missing data for the incomplete samples. Five data imputations were made in SPSS for variables with missing data points. Data were imputed using linear regression separately for each component, after which, analyses were performed as described above for the original (incomplete) dataset and the five datasets containing imputations. Effects will be reported as significant if they are present in the majority of datasets (>4 of 6), and statistics (F and P values) will be reported for the most conservative change (lowest F value).

## Results

### 3.1 Study 1 – Flip-flop Control

Raw mean amplitude data for Study 1 are available in Data S1. In Study 1, the early components, N22 and P37 were larger in response to deviant stimuli (Stimulus Type: N22 *F*_1,8_ = 11.22, *P* = 0.010; P37, *F*_1,8_ = 13.11, *P* = 0.007; Figure 2). The later component, N80, in addition to main effects of Stimulus Type (*F*_1,8_ = 37.05, *P*<0.001) and frequency (*F*_1,8_ = 8.08, *P* = 0.022), also exhibited a Stimulus Type×Frequency interaction (*F*_1,8_ = 11.06, *P* = 0.010) due to the deviant producing a larger N80 than the standard only for high frequency stimuli (*P_b_* = 0.001; *d* = 1.74). No other significant changes in the response to the deviant were identified although a similar trend-level effect was observed for N22 to the high frequency deviant (*P_b_* = 0.087; *d* = 0.65).

### 3.2 Study 2 – Many-standards Control

Raw mean amplitude data for Study 2 are available in Data S2.

#### 3.2.1 Effects of GD of Saline Treatment

In order to ensure that the pool of animals used in this study was relatively homogenous and not differentially affected by the developmental intervention at different GDs, GD was included as a between subjects factor in all statistical analyses. The only effect of GD was seen for the P30 component to high frequencies. For the P30 component, a main effect of GD (*F*_1,14_ = 5.01, *P* = 0.042) and a Stimulus Type×GD interaction (*F*_2,28_ = 3.53, *P* = 0.043) were observed. There was a significant effect of Stimulus Type for GD19 rats (*F*_2,16_ = 5.88, *P* = 0.012), but not for GD10 rats (*F*_2,12_ = 2.51, *P* = 0.122). Although not significant, the mean values for the deviant, control and standard P30 responses for the GD10s followed the expected pattern, with largest values for the response to the deviant and smallest values for the response the standard (Deviant = 7.60, Control = 6.22, Standard = 5.83). However, GD19 rats had a larger control response than both deviant and standard (Deviant = 3.72, Control = 5.02, Standard = 2.98). However, with the exception of the P30 component, overall, GD had little effect on component amplitudes and conditions. Half of the rats in Study 2 (the GD19 rats) having unexpectedly large control P30 responses relative to deviant responses may result in an overestimation of adaptation effects (Control vs. Standard) and an underestimation or reversal of deviance detection (Deviant vs. Control). This will be considered when interpreting and discussing results for P30.

#### 3.2.2 Overall Effects of Region

**Figure 3:**
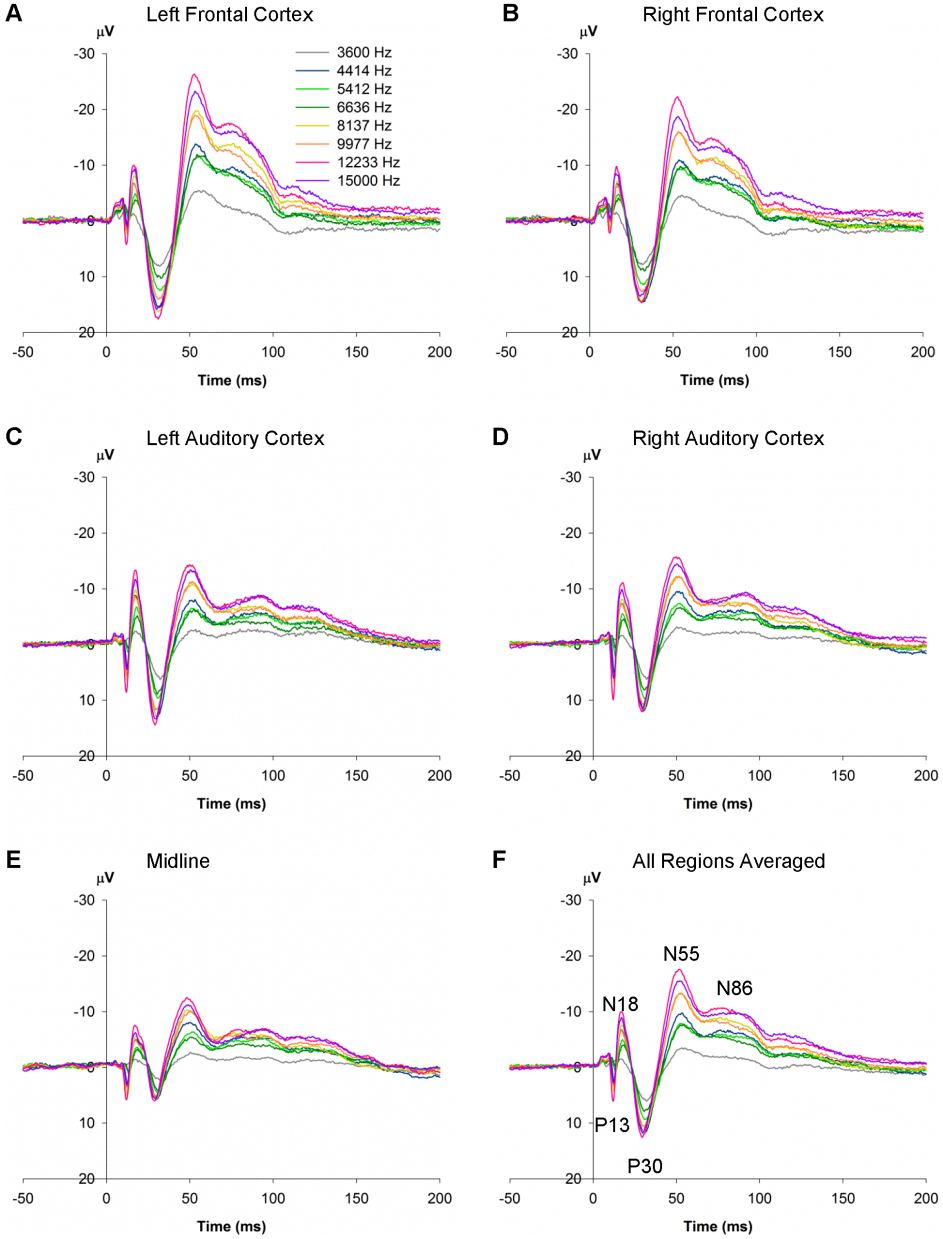
Rat ERPs in the many-standards control sequence (Study 2). ERPs recorded from electrodes implanted into the skull above the left frontal cortex (**A**), right frontal cortex (**B**), left auditory cortex (**C**), right auditory cortex (**D**), midline (**E**), and averaged over all of the regions (**F**).

Figure 3 shows the ERPs generated for each of the different frequencies in the many-standards control condition over each of the different sites, as well as for all of the sites averaged together (Figure 3F). Analysis of the effects of region were only performed on deviant and standard stimuli from the oddball sequences and the 6636 Hz and 8137 Hz (low- and high-frequency) stimuli from the many-standards control sequence. There were significant region effects for all five components. For the earliest component, P13, amplitudes were largest at the midline and auditory cortex sites and smallest at the frontal sites (*F*_3.25,21.47_ = 9.47, *P*<0.001). N18 amplitudes, on the other hand, were largest at auditory sites compared to frontal and midline sites (*F*_2.85,39.94_ = 5.33, *P* = 0.004). The later components (P30, N55 and N85) were largest at frontal cortex sites (P30: *F*_4,56_ = 25.73, *P*<0.001; N55: *F*_1.90,26.63_ = 7.50, *P* = 0.003; N85: *F*_2.05,28.75_ = 7.92, *P* = 0.002). P30 was smallest at the midline site, but both N55 and N85 exhibited equal amplitudes over auditory cortex and midline sites.

#### 3.2.3 Effects of Stimulus Type and Frequency

**Figure 4:**
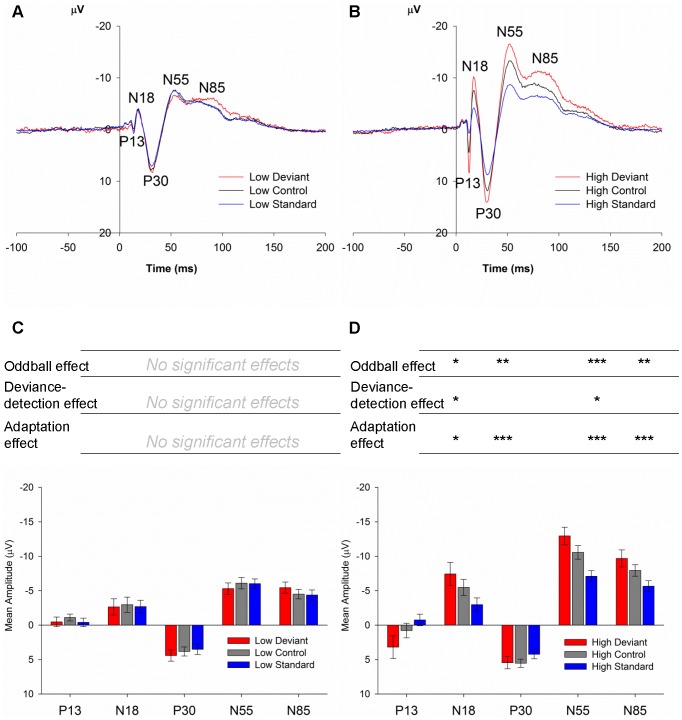
Rat ERPs in Study 2, averaged over all regions. (**A,B**) ERPs (averaged over all five regions) to the oddball deviant (red), the many-standards control (black) and the standard (blue) for the low (**A**) and high (**B**) frequency stimuli. All stimuli show a similar pattern with the same components (P13, N18, P30, N55 and N85): responses to deviants are larger in amplitude in comparison to the controls for high frequency, but not low frequency stimuli for P13 (*P_b_* = 0.023, *d* = 0.73) and N55 (*P_b_* = 0.010, *d* = 0.87). (**C,D**) Mean amplitudes (± standard error, SE) of P13, N18, P30, N55 and N85 generated by oddball deviants (red), many-standards controls (grey) and standards (blue), averaged over all five regions. Significance levels for statistical comparisons between stimulus types for each component are shown above the bars for their respective components. Asterisks indicate statistical significance under 0.05, with * 0.050<P>0.010, ** 0.010<P>0.001, *** P<0.001.

Figures 4A and 4B illustrate the ERPs to deviant, control and standard stimuli for low, and high frequency stimuli, respectively. The components P13, N18 and N55 had similar Type and Frequency effects regardless of the region recorded from. That is, while there were main effects of Region on component amplitudes, there were no interactions between Region and Frequency or Type. Therefore, the effect of Type and Frequency on these component amplitudes pooled over regions was analysed. These effects are represented in Figures 4C and 4D. A Type×Frequency effect was present for the P13 component (*F*_1.31,18.37_ = 7.16, *P* = 0.010). The effect of type was limited to high frequencies *F*_1.33,18.56_ = 10.36, *P* = 0.003. The P13 amplitude to deviant stimuli was larger than control stimuli (deviance detection, *P_b_* = 0.028; *d* = 0.73; Figure 4B,D) and standard stimuli (oddball effect, *P_b_* = 0.014; *d* = 0.85; Figure 4B,D) for high frequency stimuli, but not low frequency stimuli. In addition, P13 amplitude was larger to high-frequency control stimuli than to high-frequency standards (adaptation, *P_b_* = 0.035; *d* = 0.76; Figure 4B,D).

A similar Type×Frequency effect was observed for N18 amplitudes (*F*_2,28 = 20.90_, *P*<0.001), and again, the effect of Type was limited to the high-frequency stimuli (*F*_1.59,22.23_ = 17.30, *P*<0.001), for which N18 amplitudes to both control and deviant stimuli were larger compared to those to standard stimuli (adaptation and oddball effects, respectively; Adaptation *P_b_*<0.001; *d* = 1.33; Oddball *P_b_* = 0.001; *d* = 1.24; Figure 4B,D). Unlike the P13 component, deviance detection to the high-frequency stimuli did not reach significance for the N18 component (*P_b_* = 0.072; *d* = 0.66; Figure 4B,D).

**Figure 5:**
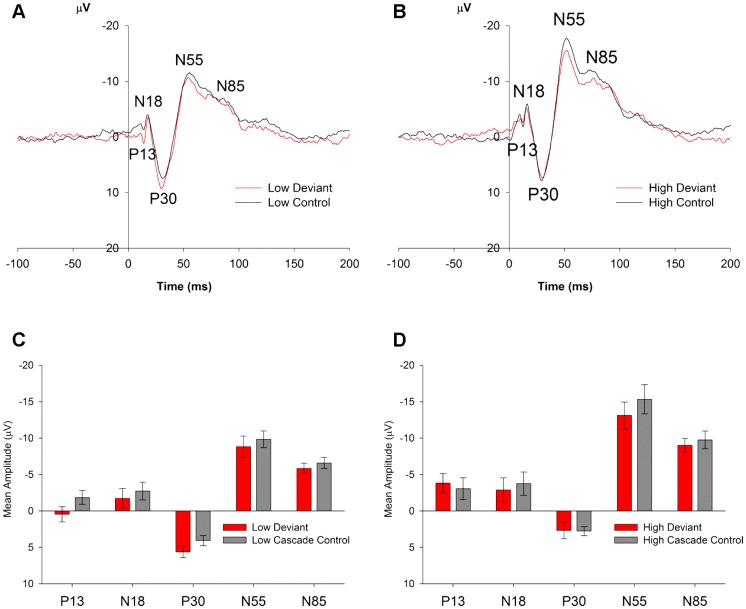
Rat ERPs in Study 3, averaged over all regions. (**A,B**) ERPs (averaged over all five regions) to the oddball deviant (red) and the cascade control (black) for the low (**A**) and high (**B**) frequency stimuli. As in Study 2, all stimuli show a similar pattern with the same components (P13, N18, P30, N55 and N85), however in this case, responses to deviants were not larger in amplitude in comparison to the controls and the standards. (**C,D**) Mean amplitudes (± standard error, SE) of P13, N18, P30, N55 and N85 generated by oddball deviants (red) and the cascade controls (grey), averaged over all five regions.

The strongest effects of Stimulus Type were observed to high-frequency stimuli for the N55 component (Type×Frequency *F*_1.68,23.53_ = 52.12, *P*<0.001), where statistically-significant oddball (*P_b_*<0.001; *d* = 2.04; Fig. 5B,D), deviance detection (*P_b_* = 0.010; *d* = 0.87; Fig. 5B,D) and adaptation effects (*P_b_*<0.001; *d* = 1.99; Fig. 5B,D) were observed. Such effects on the N55 component were not seen for low-frequency stimuli (Figure 4A,C).

For the remaining ERP components in Study 2 (P30 and N85), the recording site played a significant role in the expression of MMN-like responses (indicated by Type×Frequency×Region interactions). These effects are represented in Table 1 for all components (even though in the omnibus analysis, Region interactions were not identified for the other components, P13, N18 and N55, these are included in Table 1 for consistency).

**Table 1:** Oddball effects, Deviance detection and adaptation for each frequencies and component combination in Study 2.

		Low Frequency					High Frequency				
		Component									
		P13	N18	P30	N55	N85	P13	N18	P30	N55	N85
Oddball effect (deviant>standard)	LFC			**	*			**	**	***	**
	RFC			*						***	**
	LAC						*	**		***	**
	RAC					*	*	*		***	**
	LML						*	*		***	**
	Pooled						*	**		***	**
Deviance detection (deviant>control)	LFC				†					*	**
	RFC				†				*		**
	LAC						*			*	*
	RAC								††		
	LML						*			*	
	Pooled						*			*	
Adaptation (control>standard)	LFC							**	*	***	**
	RFC							*		***	*
	LAC							**		**	
	RAC								**	***	***
	LML							**		***	***
	Pooled						*	***		***	***

Levels of significance for the statistical difference between responses to deviant and standard stimuli (Oddball effect), deviant and control stimuli (Deviance detection) and control and standard stimuli (Adaptation) are shown for individual components and frequency conditions. Significant levels of oddball effects, deviance detection and adaptation were rarely observed for the low frequency stimuli. By contrast, high frequency stimuli often elicited both deviance detection and adaptation responses. Significance levels are indicated as: * or 0.05>P>0.01, ** 0.01>P>0.001, *** P<0.001. In the large majority of cases, the changes were in the expected direction for MMN-like effects (i.e. deviant>control>standard), indicated by unformatted asterisks. Dagger symbolss (†) indicate that the change was in the opposite direction to expected (i.e. standard>control>deviant).

A Type×Frequency×Region effect was identified for P30 (*F*_3.76,52.63_ = 5.96, *P* = 0.001). MMN-like effects were present over several regions for both high- and low-frequency stimuli. As illustrated in Table 1, the majority of MMRs for the P30 component occur over the left and right frontal sites (Deviance Detection in the RFC *P_b_* = 0.043, *d* = 0.72; Adaptation in the LFC *P_b_* = 0.001, *d* = 1.22; Oddball effects in the LFC *P_b_* = 0.004, *d* = 0.98, and RFC *P_b_* = 0.033, *d* = 0.42). No significant MMRs were observed over the midline site. At auditory sites, an adaptation effect (P30 amplitude to control >standard, *P_b_* = 0.002, *d* = 1.01) was observed for the RAC, but this was accompanied by a *reversed* deviance detection effect (i.e. amplitude to control >deviant, *P_b_* = 0.009, *d* = 0.85), and no oddball effect. These puzzling results may be driven by the interaction with GD noted in 3.2.1, where it was found that half of the rats (the rats exposed to saline at GD19), had unusually high P30 amplitudes to control stimuli. Similar to P30, N85 amplitudes were also affected by a Type×Frequency×Region interaction (*F*_5.49,76.90_ = 5.60, *P*<0.001). Oddball and adaptation effects were observed in most regions for high frequency stimuli (Table 1), but deviance detection was only observed to a statistically significant degree over the left (*P_b_* = 0.004, *d* = 1.01) and right frontal sites (*P_b_* = 0.008, *d* = 0.90) and the left auditory site (*P_b_* = 0. 039, *d* = 0.61).

### 3.3 Study 3 – Cascade Control

Raw mean amplitude data for Study 3 are available in Data S3. Contrary to Study 2, no effects of stimulus type (deviant vs. control) or stimulus type interactions were identified for Study 3 (Figure 5). GD of saline treatment did not affect responses for any of the components extracted in Study 3 (no significant main effects or interactions with GD in ANOVAs). The most prominent effects observed in Study 3 were that of region, with main effects of region being present for all components. The regional effects for each component from Study 2 were replicated in Study 3. As in Study 2, the P13 component in Study 3 was largest at the midline site and smallest at frontal cortex sites (Effect of Region *F*_4,24_ = 5.12, *P* = 0.004, observed in original data and 5/5 imputations of missing data). Similar trends were observed for the other components, all of which showed the same regional distribution of responses as in Study 2 (N18: *F*_4,52_ = 3.78, *P* = 0.009 for original data and 4/5 imputations; P30: *F*_4,24_ = 5.88, *P* = 0.002 for original data and 5/5 imputations; N55: *F*_4,24_ = 13.008, *P*<0.001 for original data and 5/5 imputations; N85 *F*_4,52_ = 6.68, *P*<0.001 for original data and 5/5 imputations).

When the imputed data were analysed for the later components (P30, N55 and N85), significant effects of Frequency were revealed for all three components. The P30 component was larger for low frequency compared to high frequency stimuli (*F*_1,13_ = 16.81, *P*<0.001 for 5/5 imputations) and the N55 and N85 components were larger for the high frequency stimuli (N55: *F*_1,13_ = 25.79, *P*<0.001; N85: *F*_1,13_ = 21.34, *P*<0.001; both for 5/5 imputations).

## Discussion

The overall aim of the current investigation was to determine optimal conditions with which to observe robust human-like MMRs in rats, with a particular focus on the type of sequence used to control for potential contributions to the size of MMR in rats. The flip-flop sequences controlled only for differences in the physical characteristics of deviant and standard stimuli but not differential adaptation. The “many controls standards” sequence (i) controlled for the probability of presentation and hence adaptation; (ii) it precluded the development of a predictive model and therefore (iii) no stimulus including the control deviant violated predictions. The cascade control sequence (i) controlled for the probability of presentation and adaptation; (ii) it provided the basis for a predictive model but (iii) the control deviant did not violate that predictive model. Significant MMRs were observed for high frequency deviants with the flip-flop design of Study 1, which as noted, did not include a control for differential adaptation. The many-standards control for adaptation, used in Study 2, replicated findings from Study 1, suggesting that the MMRs observed were due to both deviance detection and adaptation, but use of a cascade control design in Study 3, did not replicate these effects, although this control did not allow extraction of adaptation effects.

### 4.1 Oddball effects, deviance detection and adaptation

Both Study 1 and Study 2 confirmed the presence of oddball effects, that is, significant increases in ERP amplitudes in response to deviant stimuli compared to standard stimuli. While in Study 1, oddball effects were only observed to a statistically significant degree for the later negative component (N80), Study 2 using a different sound generation and recording system revealed that these oddball effects can also be observed at earlier components (P13, N18, P30), in addition to the later negative components N55 and N85. In Study 2, a many-standards control sequence was used to separate oddball effects into separate elements: an *adaptation* index, a measure of the degree of reduction in peak amplitudes to frequent standard stimuli versus control stimuli, and adaptation-independent *deviance detection* index, a measure of the degree of enhancement of peak amplitudes to deviant stimuli versus control stimuli. We therefore can separate two processes that contribute to the oddball effect: (i) deviance detection, the difference between stimuli that conform to patterns of regularity and those that defy predicted patterns and (ii) adaptation, the difference between frequently- and rarely-presented stimuli. In Study 2, by far the strongest oddball effect on peak amplitude to deviant stimuli was observed for the late N55 component, but significant effects were also observed for the earlier P13 and N18 components as well as the later N85 component. Significant levels of deviance detection and adaptation were identified for the N55 component (Figure 4D), indicating that the oddball effect on this component was driven by both adaptation-independent and –dependent processes. Similar effects were found for P13 and N85, where both deviance detection and adaptation contributed to the oddball effect, but not for N18, where only adaptation effects were observed. These findings indicate that the rat brain is capable of generating human-like MMN, and that like human MMN, these effects are in-part, independent of adaptation and driven by memory-based or prediction error signalling processes.

### 4.2 The role of frequency for MMR in rats

Studies 1 and 2 replicated the results of previous investigations in our lab [35], where deviance detection was observed for high, but not low frequency deviants when all tone frequencies were selected from a relatively low frequency range (2500–3600 Hz). In Study 1, while control for differential adaptation was not employed in this flip-flop only design, we observed similar increases in the response to the deviant, compared to the standard when tone frequencies were selected from higher frequency range (6636–8137 Hz), and closer to the optimum auditory sensitivity range of rats. The morphology of ERPs was similar to those described previously in Nakamura et al. [35], with a negative peak at approximately 20–30 ms, and a positive peak at approximately 30–40 ms (Figure 2A). Two additional negative components were identified in Nakamura et al. [35]: a negative peak at 42 ms, and a late negative difference between the deviant and the control stimuli from 50–70 ms after stimulus onset. In the current Study 1, however, these two components were replaced by a broad, and much larger negative peak from 60–100 ms. It was hypothesised that by increasing the frequency range used in Study 1 compared to Nakamura et al. [35], MMRs would also be observed for low frequency deviants. However, as in Nakamura et al., increased responses to low frequency deviants were not observed (Figure 3A and C), indicating that the lack of observable MMRs for low frequency deviants is not due to the relative sensitivity of the rat's auditory system to low frequencies, but perhaps associated with a lower salience for unexpected decreases in frequency compared to frequency increases [44]. A similar effect was also evident in Study 2. When differential adaptation was controlled for, both adaptation and deviance detection were observed in several ERP components from several sites for high-frequency deviants, but rarely occurred for low-frequency deviants. Table 1 illustrates this dramatic difference between high and low frequencies in terms of capacity to elicit oddball, deviance detection and adaptation effects. Other researchers using anesthetised rats have observed similar effects, namely evidence of MMRs to high-frequency, but not low-frequency deviants [28]. The same effect has also been observed previously for human MMN [45], as well as for changes in evoked potentials to alterations in tone frequency (increases in frequency were associated with larger ERP changes) [46]. These findings indicate an overall trend towards a higher sensitivity of the rat brain to increments in frequency rather than decrements. A possible explanation for such a trend may be that the ultrasonic vocalizations that rats use to communicate with each other are of a much higher frequency and range from 22 kHz (alarm/distress call) to 50 kHz (reward, appetitive call) [44]. The auditory system of the rat may therefore be somewhat ‘primed’ to perceive high frequency noises. Future studies could examine this by measuring MMRs to low frequency alarm (22 kHz) calls and high frequency appetitive (50 kHz) calls in a flip-flop condition. It should be noted that adaptation to low frequency changes was not observed in Study 2. While adaptation has been shown to occur for low frequency changes in other rat models [17], [21], [47], it has been found that neural populations in the rat inferior colliculus exhibit less adaptation to low frequency tones compared to high frequency tones [47].

Effects of stimulus frequency were seen for ERPs in the many standards condition (Figure 3), with increments in frequency producing larger responses (except for one frequency, 4414 Hz, which produced a larger response compared to the 5412 and 6636 Hz stimuli). As all stimuli were presented at the same intensity (70 dB_L_), the altered responses to the different frequencies could possibly be due to the sensitivity of the rat's auditory system to different frequencies. Rats exhibit low sensitivity to tones at low frequencies (<1 kHz), but this increases with increasing frequency until peak sensitivity is reached at 8 [39] to 16 kHz [40]. The current data indicate a similar effect, with responses to the 3600 Hz stimulus being relatively low amplitude, but with responses increasing in magnitude with increasing frequency, and a peak response seen to the stimuli presented at 12233 Hz, indicating that this may be the peak sensitivity range for the rats in our study. This dramatic effect of frequency on the response to different stimuli highlights the importance of controlling for stimulus identity and only comparing deviant stimuli to their respective standards and controls of the same frequency.

### 4.3 Effects of the cascade control method

Deviance detection was not observed in Study 3, which used the same recording system, electrode array and animals as Study 2, but instituted a different control method, the cascade control. Such results would indicate that contrary to Study 2, Study 3 did not find evidence of ‘true’ adaptation-independent MMN in rats. There could be a number of reasons for this lack of replication of MMRs using this method.

Firstly, the cascade control necessitated the use of a higher frequency deviant for the ascending oddball condition, in comparison to Studies 1 and 2 (15000 Hz vs. 8137 Hz). At face value, the findings from Study 3 do not conform to the suggestion proposed above, that rats are most sensitive to increments in frequency – if this is the case, one would expect to observe evidence of deviance detection for frequency increments of 12233 to 15000 Hz. It is unlikely that the lack of deviance detection to 15000 Hz stimuli is due to a lack of auditory sensitivity to the tone, because the frequency is well within the rat's frequency sensitivity range, if not at the peak of the auditory sensitivity for the rat [40]. Indeed, this peak sensitivity may be the reason why deviance detection is not observed to tones of 15000 Hz. Study 2 results revealed that in the many standards control condition (where tones from 3600–15000 Hz were presented), by far the largest ERP amplitudes (for every recording site) were observed for stimuli presented at 12233 and 15000 Hz (Figure 3), the two frequencies used as standards and deviants respectively for the ascending oddball sequence of Study 3. Therefore, perhaps the lack of deviance detection observed for deviants of 15000 Hz can be explained by a ceiling effect: the exogenous evoked potentials to both the standard and deviant tones are so large that any deviance-associated increase in the ERP simply cannot be observed. By contrast, ERP amplitudes to frequencies used as deviants and standards in Studies 1 and 2 (6636 and 8137 Hz) sit closer to the middle of the amplitude response range to differing frequencies (Figure 3), such that any amplitude changes related to deviance or adaptation are more readily observable. If indeed a ceiling effect is occurring that ‘masks’ possible effects of deviance in the cascade control condition, such effects could be minimized by shifting the range of frequencies used for the cascade control condition down (e.g. 3000 Hz–9000 Hz) so that the tones used as standards and deviants are not at the peak level of auditory sensitivity for the rat. In addition, the sound intensity of stimuli could be reduced.

Secondly, while it was suggested that the wide range of stimuli used in the many standards condition may result in an overestimation of adaptation effects [33], it has also been suggested that stimuli at the extreme ends of a range in control sequences (as used for the control deviants in the cascade control sequence) may result in control stimuli at the outer ends being perceived as deviants and again, an overestimation of adaptation in the oddball sequences [24], [42]. Therefore, the use of the cascade control sequence, with the stimuli used as deviants sitting at the outer extremes of the range of stimuli, may result in an underestimation of deviance detection. This issue would be trivial in human studies, as the pattern of regularity used in the control sequence would negate this. The third explanation for why deviance detection may not have been observed using the cascade control method is therefore that it is unknown whether the pattern of regularity established by the cascade sequence can be modelled by the rat brain. If not, higher order expectations based on these more complex statistical regularities within the environment cannot be generated and therefore, the frequencies at the extremes of the cascade sequence are as unexpected as any other of the cascade frequencies. In fact, given that the extreme frequencies occur with a lower probability than other cascade frequencies (12.5% vs. 25%), in the absence of a rule that governs the cascade sequence regularity, they will appear to be aberrant (rare) and therefore generate deviance detection in their own right. In addition, without data from a flip-flop control standard being measured, it is not known whether the animals in Study 3 are even exhibiting any oddball response (a larger response to the deviant vs. the standard. The results from Study 3 therefore remain somewhat inconclusive. Future studies should include four oddball sequences for the cascade control method – the two used in the current study, as well as flip-flop controls for each of them. In addition, further examination of the cascade control, how it compares to the many standards control, and the ability of the rat brain to model such complex regularities is warranted.

### 4.4 Relationship of these data to MMRs in humans

The results from Study 2 revealed that MMRs (both adaptation and deviance detection) can be observed in the late, negative components, N55 and N85, which most closely resemble human MMN in their polarity (negative) and their relative latency (it is expected for ERP components to occur with a reduced latency in the rat brain [48]). However, oddball effects were also observed on earlier components such as P13 and N18 (and P30 to a lesser degree). This may seem at variance with the human MMN literature that has focussed on late effects. However, recent research has shown evidence of adaptation-independent deviance detection on human middle latency responses (MLRs). In human investigations of MMN, a bandpass filter (e.g. 0.1–35 Hz) is typically applied, which filters out early high frequency midlatency ERP components, but allows the slower MMN component to be observed. However, when a suitable bandpass filter is applied so that early ERPs can be detected (e.g. 15–200 Hz), additional MLR components can be observed [49]. These include positive peaks at approximately 12 and 30 ms (P0 and Pa) and negative peaks at approximately 22 and 40 ms (Na and Nb) [49]. Several human studies have now confirmed evidence of deviance detection in MLRs, notably Na peaking at 20 ms [50], Pa at 30 ms [51], and Nb peaking at 40 ms [49]. The bandpass filters associated with our data acquisition system permitted the detection of a series of early responses that exhibited adaptation and/or deviance detection. These early components might be homologues for the human MLR components that show deviance detection, although further research is required to support this view.

This study also highlights the importance for including controls for differential adaptation in human studies, which rarely occurs (for review, see [2]). While it is known that MMN in healthy subjects includes an adaptation-independent deviance detection component, we thus far do not know if observed reductions in MMN (for example, in patients with schizophrenia, or in subjects given NMDAR antagonists [2]), are due to reductions in adaptation or deviance detection, a very important question for future research into the functional importance of MMN in disease states [2].

### 4.5 The role of recording site in component amplitudes and the expression of adaptation and deviance detection

Studies 2 and 3 recorded ERPs from five separate sites over the rat cortex: over left and right auditory cortices (LAC and RAC), left and right frontal cortices (LFC and RFC) and another at the midline, similar to the vertex in human recordings (due to the bone suture at the exact midline the electrode was placed just left of the midline, LML). Marked effects of site were found for the amplitudes of all ERP components in Studies 2 and 3 (Figure S1). P13, the earliest component, was largest at the midline and auditory sites and smallest at frontal sites. This pattern of scalp topography contrasts with the later components, which were smallest at the midline site. N18 was largest at the auditory sites and the remaining later components (P30, N55 and N85) were maximal at frontal sites.

The site of the recording electrode also had a significant impact on the expression of rat MMRs for P30 and N85, but not P13, N18 and N55. MMRs were rarely observed for P30, but tended to be over the frontal cortex and auditory cortex sites, not midline sites (where P30 was smallest). Similarly, deviance detection at N85 was observed only for the sites where N85 was largest (both frontal sites and LAC site). These findings do not necessarily indicate that MMRs can only be observed at particular locations, but rather suggest that the capability of detecting statistically significant changes at a particular recording site or region is reliant on that site producing a strong signal. Since deviance detection was strongest for the N55 and N85 components, and these components are largest at frontal cortex sites, recording from frontal sites is most likely the best choice for observing human-like deviance detection in the rat.

### 4.6 Conclusions

This study presents a careful characterisation of different control paradigms, stimulus frequencies and recordings sites and how readily they detect MMN-like responses in the rat brain. The data presented in this study contribute to a growing body of evidence [28], [29], [32], [34]–[38] supporting the conclusion that the rat brain is quite capable of producing MMRs that are similar to the human MMN and are not entirely dependent on neural adaptation but rather are in part, contributed to by a more complex deviance detection process. This model can now be used to investigate the neurobiology of both adaptation and adaptation-independent deviance detection, using different pharmacological, developmental and neurobiological manipulations.

## Supporting Information

Figure S1 **ERPs in rats to deviant, control and standard stimuli for low and high frequency conditions in Study 2 for each region.** ERPs for each of the five regions recorded from to the oddball deviant (red), the many-standards control (black) and the standard (blue) for the low (left) and high (right) frequency stimuli. All stimuli show a similar pattern with the same components (P13, N18, P30, N55 and N85).
(TIF)

Data S1 **Mean amplitude data from Study 1.** Data values are mean amplitudes (in µV) of the components N22, P37 and N80, measured in Study 1. ‘High’ or ‘Low’ in the variable name refers to whether the ERP response was to the high frequency or low frequency stimulus. ‘Dev’ or ‘Std’ refers to whether the ERP response was to a deviant or standard stimulus, respectively.
(XLSX)

Data S2 **Mean amplitude data from Study 2.** Data values are mean amplitudes (in µV) of the components P13, N18, P30, N55 and N85, measured in Study 2. ‘High’ or ‘Low’ in the variable name refers to whether the ERP response was to the high frequency or low frequency stimulus. ‘Con’, ‘Dev’ or ‘Std’ refers to whether the ERP response was to a control, deviant or standard stimulus, respectively. ‘GD’ is the gestational day of saline exposure. Recording sites are identified in the variables by the following abbreviations: AvReg – “Averaged region”, mean amplitude averaged over all regions; LAC, Left auditory cortex; LFC, Left frontal cortex; LML, midline (slightly to the left of); RAC, Right auditory cortex; RFC, Right frontal cortex.
(XLSX)

Data S3 **Mean amplitude data from Study 3.** The data for the mean amplitudes (in µV) of each component of the ERPs from Study 3 (P13, N18, P30, N55, N85) are represented on separate sheets of the spreadsheet file. Incomplete, original data are labelled as imputation = 0, the imputed data are labelled as imputations 1–5. ‘High’ or ‘Low’ in the variable name refers to whether the ERP response was to the high frequency or low frequency stimulus. ‘Con’, ‘Dev’ or ‘Std’ refers to whether the ERP response was to a control, deviant or standard stimulus, respectively. ‘GD’ is the gestational day of saline exposure. Recording sites are identified in the variables by the following abbreviations: AvReg – “Averaged region”, mean amplitude averaged over all regions; LAC, Left auditory cortex; LFC, Left frontal cortex; LML, midline (slightly to the left of); RAC, Right auditory cortex; RFC, Right frontal cortex.
(XLSX)
