# Supplementary figures and images for: Mismatch Negativity (MMN) in Freely-Moving Rats with Several Experimental Controls
Source: PLoS One. 2014 Oct 21;9(10):e110892. doi: 10.1371/journal.pone.0110892 (PMC4205004; doi:10.1371/journal.pone.0110892)

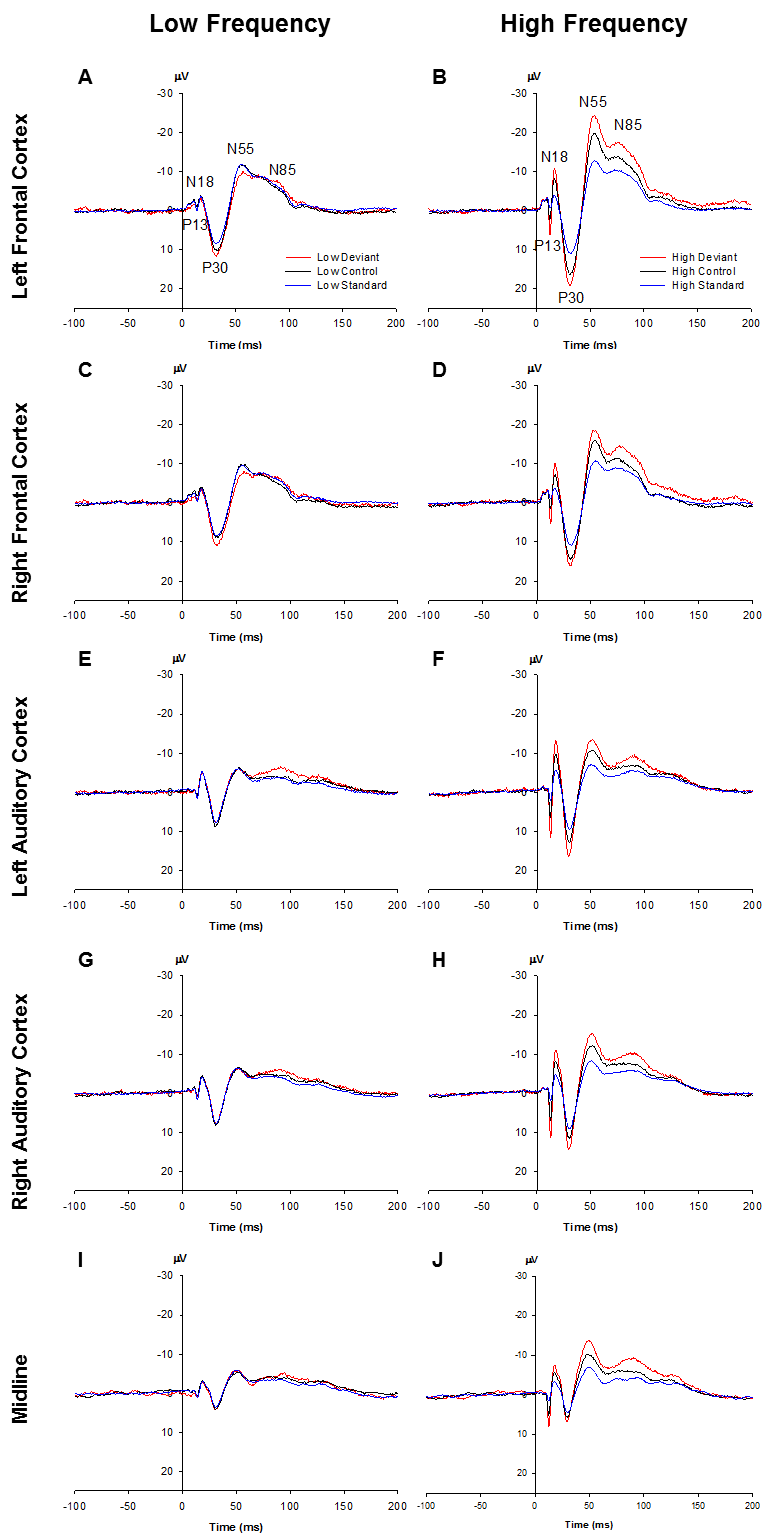

Supplement: Figure S1 — ERPs in rats to deviant, control and standard stimuli for low and high frequency conditions in Study 2 for each region. ERPs for each of the five regions recorded from to the oddball deviant (red), the many-standards control (black) and the standard (blue) for the low (left) and high (right) frequency stimuli. All stimuli show a similar pattern with the same components (P13, N18, P30, N55 and N85). (TIF) [file pone.0110892.s001.tif]
